# Differences in Gene Expression Profiles of Seven Target Proteins in Third-Stage Larvae of *Anisakis simplex* (Sensu Stricto) by Sites of Infection in Blue Whiting (*Micromesistius poutassou*)

**DOI:** 10.3390/genes11050559

**Published:** 2020-05-17

**Authors:** Marialetizia Palomba, Paolo Cipriani, Lucilla Giulietti, Arne Levsen, Giuseppe Nascetti, Simonetta Mattiucci

**Affiliations:** 1 Department of Public Health and Infectious Diseases, Section of Parasitology, Sapienza University of Rome, 00185 Rome, Italy; marialetizia.palomba@uniroma1.it; 2 Section of Contaminants and Biohazards, Institute of Marine Research (IMR), P.O. Box 1870 Nordnes, 5817 Bergen, Norway; paolo.cipriani@hi.no (P.C.); lucilla.giulietti@hi.no (L.G.); arne.levsen@hi.no (A.L.); 3 Department of Biological and Ecological Sciences, Tuscia University, 01100 Viterbo, Italy; nascetti@unitus.it

**Keywords:** *Anisakis simplex* (s.s.), gene expression, infected tissues, fish host, *Micromesistius poutassou*

## Abstract

The third-stage larvae of the parasitic nematode genus *Anisakis* tend to encapsulate in different tissues including the musculature of fish. Host tissue penetration and degradation involve both mechanic processes and the production of proteins encoded by an array of genes. Investigating larval gene profiles during the fish infection has relevance in understanding biological traits in the parasite’s adaptive ability to cope with the fish hosts’ defense responses. The present study aimed to investigate the gene expression levels of some proteins in L3 of *A. simplex* (s.s.) infecting different tissues of blue whiting *Micromesistius poutassou*, a common fish host of the parasite in the NE Atlantic. The following genes encoding for *Anisakis* spp. proteins were studied: Kunitz-type trypsin inhibitor (*TI*), hemoglobin (*hb*), glycoprotein (*GP*), trehalase (*treh*), zinc metallopeptidase 13 (*nas 13*), ubiquitin-protein ligase (*hyd*) and sideroflexin 2 (*sfxn 2*). Significant differences in gene transcripts (by quantitative real-time PCR, qPCR) were observed in larvae located in various tissues of the fish host, with respect to the control. ANOVA analysis showed that relative gene expression levels of the seven target genes in the larvae are linked to the infection site in the fish host. Genes encoding some of the target proteins seem to be involved in the host tissue migration and survival of the parasite in the hostile target tissues of the fish host.

## 1. Introduction

Third-stage nematode larvae (L3) of the genus *Anisakis*, mainly *Anisakis simplex* (s.s.), infect several marine species of the Arctic–Boreal Region [1]. Planktonic or semi-planktonic crustaceans are intermediate hosts; fish and squids act as paratenic/intermediate hosts, while cetaceans are definitive hosts in the life cycle of these parasites [1,2]. Fishes acquire *Anisakis* spp. larvae through the diet, preying upon infected crustaceans and/or other fish species. Once infected prey is digested, the larvae are able to penetrate the stomach wall of the fish [3] and migrate into the visceral cavity. After crossing the stomach wall, *Anisakis* spp. larvae usually settle on the external surface of internal organs, such as liver, gonads, and mesentery, eventually followed by host-generated encapsulation. However, some larvae may migrate *intra-vitam* or *post-mortem* into the body musculature of the fish host [4,5,6,7,8,9].

*Anisakis* spp. third-stage larvae are considered parasites of generally low pathogenicity and virulence in fish [3]. However, during tissue migration, the larvae can modify the structure and function of the host tissues, causing hemorrhages and focal immune reactions [10]. The severity of the pathological effects differs widely, depending on the intensity of infection and the parasitized tissue [10,11,12,13]. Moreover, various fish host species may show differential susceptibility to the infection [10], and considerable differences seem to exist between fish species with respect to their ability to respond, immunologically, against the larvae [3,10]. *Micromesistius poutassou* is apparently not associated with any significant tissue damage, unlike the Atlantic mackerel, which appears to be capable of reducing the *A. simplex* (s.l.) infection by immunological means [3]. Additionally, the infection-site selection of the L3 would differ with both fish species and species of *Anisakis* [1,10]. 

In an experimental study, Bahlool et al. [13] elucidated the influence of larval excretory/secretory (ES) products on the fish immune system by measuring the immune gene expression in spleen and liver of rainbow trout (*Oncorhynchus mykiss*) in response to an intraperitoneal injection of excretory/secretory (ES) products isolated from L3 of *A. simplex* (s.l.). The overall gene expression profile of the injected hosts showed a down-regulation of certain immune genes, suggesting that ES products from the nematode larva can dampen the immune reactions of fish [13,14]. Recently, Marnis et al. [15] also showed a worm-induced immune suppression (i.e., downregulation of genes encoding cytokines) locally in the infected liver of the Baltic cod (*Gadus morhua*) with *Contracaecum osculatum*. However, some ES products seem also to be able to elicit immune responses, and their immunogenicity can provoke antibody production [16,17,18,19,20].

The analysis of transcribed mRNAs of *Anisakis* spp. larvae can provide useful information on the genes associated with the parasite–host interactions [21]. In particular, gene expression profiles of adaptive molecules may provide clues related to their role(s) in the biological pathways and pathogenesis of L3 *Anisakis* spp. in naturally and accidentally infected hosts (human), and it remains an aspect of the host–parasite interaction to be further investigated [22,23,24,25]. According to Palomba et al. [25], temperature can play an active role in modulating the gene expression profiles of immunogenic and adaptive proteins (i.e., the Kunitz-type trypsin inhibitor and the hemoglobin) in L3 of *A. pegreffii*. Likewise, a down-regulation of the glycoprotein (*GP*) gene expression was found under different temperature conditions, suggesting a possible role of other factors, such as the host immune responses [25]. In this respect, one may assume that during the penetration process as well as after having settled in or on a target organ, the L3-larva transcribes mRNAs related to different biological processes (e.g., invasion, migration, and survival). 

Thus, the present study aimed to evaluate the gene expression profiles of some proteins of the parasite species *A. simplex* (s.s.) in different tissues of a naturally infected fish host, the blue whiting (*Micromesistius poutassou*). Target genes were selected among those coding for proteins which have previously found by other authors to be highly expressed in L3 of *Anisakis* sp. [21], either up- or down-regulated under the effect of various abiotic conditions [25], as well as found as up-regulated in the transcripts of the larval pharyngeal tissues of *A. simplex* (s.s.) [23]. In addition, the herein investigated genes on the one hand are known to be involved in facilitating parasite survival and adaptation to the host, and on the other, they are able to trigger the host immune response [21,24,26].

## 2. Materials and Methods 

### 2.1. Anisakis spp. Larvae Sampling 

A total of *N* = 60 blue whiting (total mean length 280 ± 35 mm) were sampled in April 2018 off St. Kilda (N 58°04′ W 09°40′), in the NE Atlantic Ocean (FAO 27 area, Division VI a, Northwest Coast of Scotland and North Ireland or West of Scotland) onboard the commercial fishery and research vessel MS *Kings Bay* (Institute of Marine Research cruise no. “Kings Bay” 2018843). Fish were freshly analyzed onboard, and *Anisakis* spp. L3 were collected within one hour post-catch. The infection site of each larva was recorded. A total of *N* = 27 larvae, nine from each site of infection (i.e., from viscera (mesenteries), liver and muscle), were rinsed several times in sterile saline solution (0.9% NaCl) and stored in RNA*later*^TM^ (Sigma-Aldrich, St. Louis, MO, USA) (24 h at 4 °C, then at −20 °C) for further molecular identification and gene expression analysis. A set of *Anisakis* spp. larvae (*N* = 9) recovered from the fish stomach were used as control in the quantitative real-time PCR (qPCR) assay. Larvae collected from the stomach were considered as just acquired by the fish during predation upon other infected intermediate/paratenic hosts and mainly from amphipods, appendicularians, and euphausiids [27] (i.e., larvae not migrated). 

### 2.2. Total DNA/RNA Isolation and cDNA Synthesis 

DNA and RNA were simultaneously extracted from each *Anisakis* larva using TRIzol reagent (Invitrogen, Carlsbad, CA, USA) according to the manufacturer’s instructions, with some modifications, as described in [25]. Briefly, each larva was homogenized in 1 mL of TRIzol. After the sample was homogenized, 0.2 mL of chloroform was added to separate the aqueous phase containing RNA from the interphase/organic phase containing DNA. 

DNA was precipitated from the interphase/organic layer with 300 μL of ethanol, washed several times to remove impurities and then resuspended in 40 uL of nuclease and RNase-free water. DNA was stored at −20 °C for the larval identification at species level, based on the direct sequence analysis of mitochondrial (mtDNA *cox2*) and nuclear (elongation factor EF1 α-1 of nDNA) gene loci.

RNA was precipitated from the aqueous layer with 0.5 mL of isopropanol, washed and then resuspended in 40 μL of nuclease and RNase-free water. Total RNA was subsequently treated with DNase (DNase I, Invitrogen) to remove any genomic DNA contamination, according to the manufacturer’s protocol. RNA concentration and purity were determined by spectrometry using a NanoDrop^®^TC1-E20 spectrophotometer (BioTek Synergy^TM^ HT, BioTek Instruments, Winoosky, VT, USA) and by 1.5% agarose gel electrophoresis. RNA was reverse-transcribed to cDNA using High-Capacity cDNA Reverse Transcription (RT) kit (Applied Biosystems, Foster City, CA, USA) from an RNA pool of three larvae of *Anisakis simplex* (s.s.) previously identified, to increase the efficiency of normalization and maximize the number for which quantitative data were obtained. 

The RT reaction was carried out at 37 °C for 2 h. A final volume of 20 μL of cDNA was stored at –20 °C for further Real-Time qPCR analysis.

### 2.3. DNA Sequencing for Larval Identification

The *Anisakis* spp. larvae used in the gene expression experiments (*N* = 36) were identified at species level by means of genetic/molecular markers. For this purpose, a multi-locus approach based on the sequences’ analysis of mitochondrial (mtDNA *cox2*) (629 bp) and nuclear (EF1 *α*-1 nDNA) genes was applied.

For sequencing the mitochondrial cytochrome C oxidase subunit II (*cox2*) gene, PCR amplification was performed using the primers 211F (5′-TTTTCTAGTTATATAGATTGRTTTYAT-3′) and 210R (5′-CACCAACTCTTAAAATTATC-3′) [28]. Polymerase chain reaction (PCR) was carried out according to the procedures provided by Mattiucci et al. [28]. The sequences obtained at the mtDNA, for the *Anisakis* spp. larvae, were compared with those previously deposited in the GenBank database. 

All the *Anisakis* spp. larvae, identified by the mtDNA *cox2* gene, were sequenced at the elongation factor (EF1 *α*−1 nDNA) nuclear gene. The EF1 *α*−1 nDNA was amplified using the primers EF-F (5′-TCCTCAAGCGTTGTTATCTGTT-3′) and EF-R (5′-AGTTTTGCCACTAGCGGTTCC-3′) [29]. The PCR conditions and procedures followed those previously reported in [29]. The sequences obtained for the EF1 *α*−1 nDNA gene for the larval specimens were compared with those previously deposited in GenBank, at the diagnostic positions (i.e., 186 and 286) as previously detailed [29]. 

### 2.4. Gene Expression by Quantitative Real-Time PCR

Quantitative real-time PCR (qPCR) was carried out using the StepOnePlus^TM^ system (Applied Biosystems) to detect and quantify the transcript amounts of *TI* (Kunitz-type trypsin inhibitor), *hb* (hemoglobin), *GP* (glycoprotein), *treh* (trehalase), *nas 13* (zinc metallopeptidase 13), *hyd* (ubiquitin-protein ligase), and *sfxn 2* (sideroflexin 2), in *A. simplex* (s.s.) larvae recovered from different sites of infection of the blue whiting.

Reactions were performed in triplicates, and the protocol used was optimized for a total reaction volume of 20 µL: 0.5 µL forward and reverse primers (10 µM each) (Table 1), 10 µL of SYBR^TM^ Green PCR Master Mix (Applied Biosystems) containing SYBR Green I dye, AmpliTaq Gold DNA polymerase, dUTP and buffer, 1.0 µL of 10-fold diluted cDNA and 8.0 µL of dH_2_O.

Primers used for the *TI*, *GP,* and *hb* were previously designed by Palomba et al. [25], while those for *sfxn 2*, *hyd*, *treh,* and *nas 13* genes were designed by Kim et al. [21]. Gene expression results were normalized to the geometric mean of *gpd* (Glyceraldehyde-3-phosphate dehydrogenase), used as reference gene [30] (Table 1). The qPCR assays conditions were the following: one cycle at 95 °C for 10 min, 40 cycles of a denaturation step at 95 °C for 15 s, and annealing step at 57–60 °C for 1 s (Table 1). Melting curve data were collected at 55–95 °C. 

Gene expression data were analyzed according to the ΔΔCt method [31]. Change in threshold cycle (ΔCt) was calculated as the difference between the target gene and the reference gene for each sample. ΔΔCt was calculated as the difference between the ΔCt of the control group and the ΔCt of the experimental groups. 

### 2.5. Statistical Analyses 

Statistical analyses were performed by using GraphPad Prism version 6.0 (GraphPad Software, La Jolla, CA, USA, www.graphpad.com). Normality was analyzed using the Shapiro–Wilk test. Statistical differences between the gene expression levels of *Anisakis* spp. larvae by fish host tissues were assessed by applying one-way ANOVA followed by Dunnett’s multiple comparison test to compare the mean of each site of infection with the mean of the control group and between the sites of infection. 

Significant differences were considered at *p <* 0.05 for all statistical tests performed. Data are presented as mean (M) ± standard deviation (SD). Significant differences are indicated with different asterisks.

## 3. Results

### 3.1. Identification of Anisakis spp. 

According to the sequences of 629 bp in length of the mtDNA *cox2* gene locus [29], all the *Anisakis* spp. larvae used in this study were assigned to the species *A. simplex* (s.s.). The sequences obtained in the present study matched 99%–100% with the sequences previously deposited in GenBank for the species. In addition, the identity of the specimens recognized by mtDNA *cox2* seq. analysis as belonging to *A. simplex* (s.s.) was confirmed by analysis of the two diagnostic nucleotide positions observed at the locus EF1 *α*-1 nDNA [29]. 

### 3.2. qPCR Analysis of Gene Coding for Seven Target Proteins

**Figure 1:**
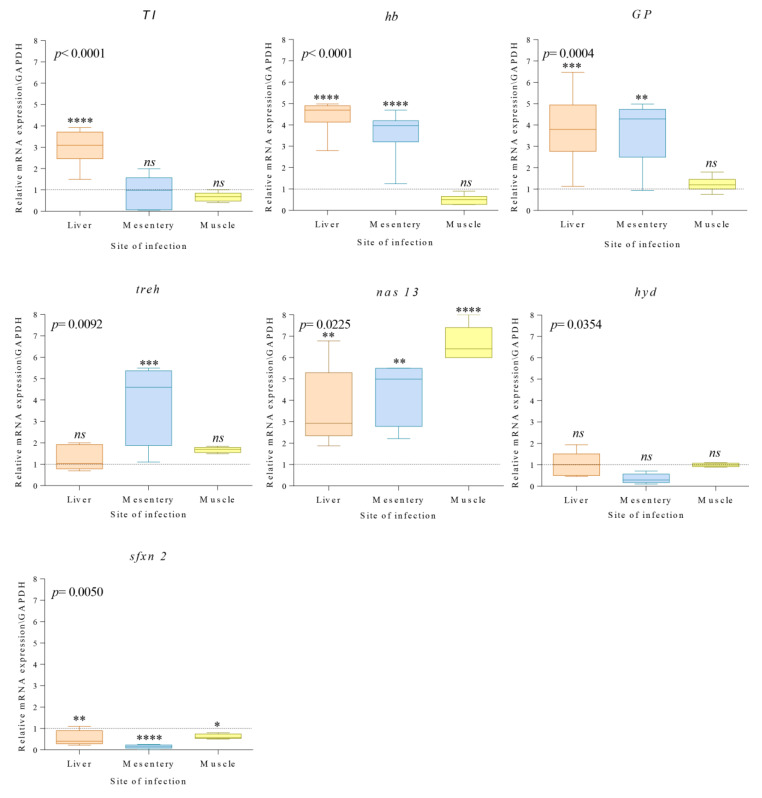
Box-plot representation of relative expression profiles of 7 genes coding for proteins, i.e., *TI, hb, GP, treh, nas 13*, *hyd, sfxn 2,* normalized to the geometric mean of *gpd* in *Anisakis simplex* (s.s.) larvae infecting different host-fish tissues (liver, mesentery, muscle). The control, represented by larvae recovered in the stomach lumen of the fish host, is given as normalized to a value of 1 (dashed line). Each box shows the minimum, first quartile, median, third quartile, and maximum gene expression values of three biological replicates. Asterisks indicate the significance level of the differences between each group of *A. simplex* (s.s.) larvae from different sites of infection and the control group. Significance was fixed at *p* < 0.05 (* *p* < 0.05, ** *p* < 0.01, *** *p* < 0.001, **** *p* < 0.0001); *ns* = not significant. One-way ANOVA analysis on the relative gene expression is shown in the top left of each box.

The analysis of gene expression profiles of seven adaptive proteins of the L3 of *A. simplex* (s.s.) recovered from different sites of the parasite infection (liver, mesentery, muscle) in *M. poutassou* are shown in Figure 1. 

Quantitative real-time PCR (qPCR) analysis revealed differential regulation of the target genes in response to the infection site of the *A. simplex* (s.s.) larvae. Shapiro–Wilk test supported a normal distribution of the data obtained from the qPCR assay tests (all tests *p* < 0.05). Comparison in the expression levels of genes coding for Kunitz-type trypsin inhibitor, hemoglobin, glycoprotein, trehalase, zinc metallopeptidase 13, ubiquitin-protein ligase, and sideroflexin 2 was performed between *A. simplex* (s.s.) larvae collected from different sites of infection and the control (Figure 1). 

The gene coding for the Kunitz-type trypsin inhibitor in the *A. simplex* (s.s.) larvae located in the liver showed statistically significant higher levels of expression (*p <* 0.0001) (Figure 1) compared to larvae of the control (stomach lumen). No significant differences were recovered for the same gene in *A. simplex* (s.s.) larvae located in the mesentery and muscle (*p =* 0.571, *p =* 0.301, respectively) (Figure 1). 

A statistically significant up-regulation of the *hb* gene, coding for the hemoglobin, was observed in *A. simplex* (s.s.) larvae recovered from the liver (*p <* 0.0001) and mesentery (*p <* 0.0001) (Figure 1). Standard levels of the *hb* transcripts (*p =* 0.208) were measured in the larvae located in the muscle with respect to the control. 

The *GP* gene was found to be significantly up-regulated in *A. simplex* (s.s.) larvae collected from the liver (*p <* 0.0001) and mesentery (*p =* 0.0015), while the expression values of this gene in larvae from the muscle were at a level similar to the control (*p =* 0.760). 

No significant difference was recorded in the expression levels of the *treh* gene coding for the trehalase between *Anisakis* larvae collected from the fish liver (*p =* 0.596) and muscle (*p =* 0.230) and the control. Conversely, significant (*p =* 0.0002) were the differences in the transcripts of this gene estimated in the parasites recovered from mesenteries.

The *nas 13* gene of *A. simplex* (s.s.) showed a generally significant (*p <* 0.05) up-regulation of the larvae located in all of the infection sites compared to the larvae of the control group (Figure 1). 

A low gene expression level was recorded for the *hyd* gene coding for the ubiquitin-protein ligase, studied in the larvae from liver, mesenteries, and muscle of the fish host; nevertheless, non-significant differences *(p >* 0.05) were recorded in comparison with the larvae of the control. 

Finally, the transcripts levels of the *sfxn 2* gene coding for the sideroflexin 2 were significantly down-regulated (*p <* 0.05) in *A. simplex* (s.s.) larvae located in all fish tissues.

Dunnett’s multiple comparison test for differences in expression level per target gene between larvae from different fish host tissues are shown in Table 2. The ANOVA analysis showed that the overall transcripts values of the seven selected genes in *A. simplex* (s.s.) were significantly (*p <* 0.05) affected by the infection site of the parasite species in the fish host (Figure 1).

## 4. Discussion

In the present study, target gene transcripts of L3 of *A. simplex* (s.s.), correlated with their tissue localization in a commonly infected fish host, have been investigated for the first time.

The selected fish host species, blue whiting, is an intermediate/paratenic host in the life cycle of *A. simplex* (s.s.), which acquires the parasite through predation on other infected hosts (i.e., crustaceans, small fish, and cephalopods) [27,32]. The fish host populations caught in the NE Atlantic Ocean from off St. Kilda island (FAO VIa) are known to have high infection levels of this parasite both in the liver, mesenteries, and flesh [1,33,34]. Moreover, blue whiting from the present fishing area are usually not infected with other anisakid nematode species [34,35,36], which, in this respect, makes this fish host a suitable model since interference from other closely related anisakid species is not likely to occur.

*Anisakis simplex* (s.s.) larvae usually penetrate the gastric wall of the fish host to reach the visceral cavity. There, some L3 settle on the surface of the visceral organs (i.e., mesentery, liver, gonads), followed by host-induced encapsulation, where the capsule thickness seems largely to depend on infection site, i.e., the host target organ [3,37]. However, some larvae may migrate into the musculature of fish or squid [1,4,8,34,38,39], but so far, only L3 of *A. simplex* (s.s.) and *A. pegreffii* have been recorded in the muscular tissue of various fish and squid hosts, with *A. simplex* (s.s.) having apparently a higher capability to invade the flesh of fish than its sibling species *A. pegreffii* [7,33,40,41]. Once established and encapsulated, the larvae go through a period of latency, remaining infective until the next host (i.e., another transport host or a definitive marine mammalian host) [2]. To date, few studies have investigated the interactions between *Anisakis* spp. and their intermediate/paratenic hosts. Levsen and Berland (personal observation, 2012) observed that different fish hosts might show different degrees of immunological reactions to larvae. In some fish hosts, including the target species of this study, *M. poutassou*, evident melanomacrophage aggregates around larval infections sites on the liver have been observed [3]. The role of these aggregates is not yet completely understood, but Agius and Roberts [42] suggested that the melanomacrophage aggregates might develop in association with late-stage chronic inflammations induced by parasites. Recently, a cellular immune response against *A. simplex* (s.s.) larvae was observed in the liver of blue whiting specimens of the same samples examined in the present study; conversely, no host reaction was present around larvae lodged in the musculature of fish (Dezfuli pers. obs.).

Larsen et al. [43] assumed that the parasite-secreted molecules could be the cause of fish host cellular reactions. 

In other studies, the gene expression of target proteins of L3 *A. simplex* (s.l.) was compared with L4 stage larvae [21]. Additionally, the *A. pegreffii* L3 gene expression at different temperature conditions was tested and compared [25,30]. However, no study concerning the differential gene expression related to the *Anisakis* spp. fish–host interaction has so far been carried out.

**Figure 2:**
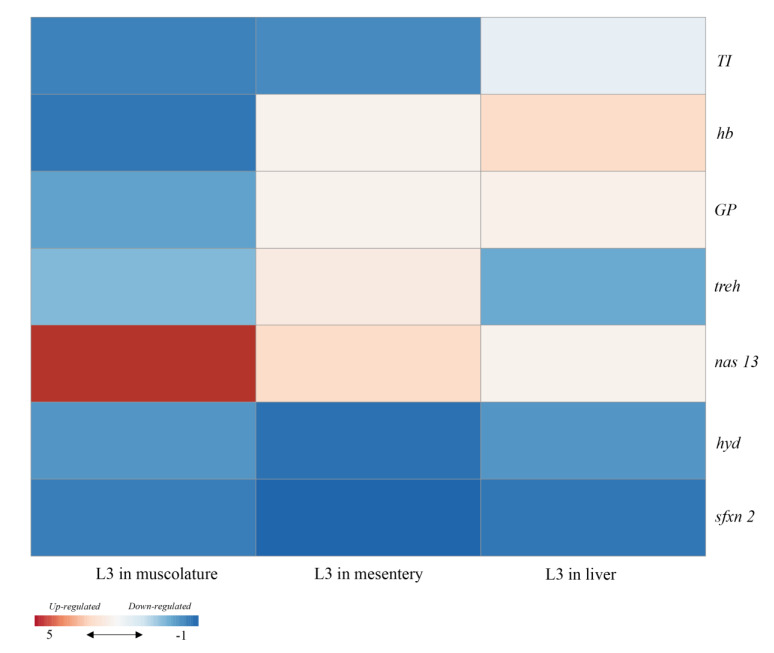
Heat map presenting the changes in the gene expression of *Anisakis simplex* (s.s.) larvae collected from different fish tissues. Gene expression was first calculated relative to the geometric mean of *gpd* and then was standardized following a standard score normalization against the corresponding control (larvae recovered in the internal lumen of the stomach) samples. Shades of red and blue, respectively, indicate the highest and lowest expression levels, as specified in the scale bar at the bottom of the figure. This heatmap was produced with the ClustVis web tool [83].

In this study, amongst the selected target genes, a significant differential gene expression of the *TI* gene coding for a Kunitz-type trypsin inhibitor protein was detected in *A. simplex* (s.s.) larvae removed from the fish liver (Figure 2). The Kunitz-type trypsin inhibitor protein seems to have important functions in the parasite’s development [44,45] and in the host proteases’ inhibition [20,46]. Indeed, a wide range of diverse nematode species has been found to express proteins with trypsin-inhibitor-like domains, including *Ascaris suum* [47], *Ancylostoma caninum* [48], and *Trichuris suis* [44]. Many of these nematode proteins have been shown to inhibit serine proteases, e.g., chymotrypsin, trypsin, elastase, and, in the case of the hookworms, host’ thrombin and coagulation factor [49,50,51]. Hawley and Peanasky [52] assumed that, in order to parasitize a host, a nematode requires those protease inhibitors that interact strongly with host proteases located in different sites of infection. The Kunitz-type trypsin inhibitor protein would aid *A. simplex* (s.s.) to counteract the host proteolytic enzyme and coagulation factors. Indeed, proteins with Kunitz domains have previously been detected in expressed transcripts sets obtained from pharyngeal tissues of *A. pegreffii* and *A. simplex* (s.s.) larvae [22,23]. In this study, an up-regulation of transcripts of *TI* in *A. simplex* (s.s.) located in the liver could be correlated to the fish host’s proteolytic enzymes, i.e., serine proteases. Fish organs are a source of those enzymes, especially digestive proteases, such as aspartic protease pepsin and serine proteases (i.e., trypsin, chymotrypsin, and elastase) [53,54]. In addition, some serine proteases, such as the tryptases, are produced and stored in the mast cell granules located in different host immune tissues [55]. Those host serine proteases would be implicated in the response to invading parasite larva which again could react with increasing production of inhibitor proteins, e.g., the Kunitz-type trypsin.

Nematode hemoglobins are molecules with an unusually high oxygen affinity. Several studies indicate that they may represent an important component of parasite adaptation to the co-existence with the host [25,56,57,58]. Thus, hemoglobin, sequestering oxygen, has been suggested to aid the parasite in maintaining a locally anaerobic environment, while their ability to break down nitric oxide (NO) and hydrogen peroxide produced by innate immune cells would also aid the parasite survival. Hemoglobin’s catalase and nitric oxide dioxygenase activities are important in protection against host immune responses to infection [59]. Results obtained in this study seem to support the possible function of the parasite’s hemoglobin in increasing its protection against the host immune response. Cavallero et al. [23] reported the presence of transcripts of *hb* even in pharyngeal tissues of *Hysterothylacium aduncum* larvae infecting viscera of the fish host. An up-regulation of the gene *hb* is reported as well in the present study for the larvae located in both host viscera (i.e., mesentery) and liver (Figure 2).

In previous investigations [25], we found that since the transcript levels of the *GP* in *A. pegreffii* remained stable under certain temperature conditions, its gene expression level would be modulated by the host immune response. Indeed, we observed in the present study that the transcripts of a *GP* gene were significantly up-regulated in *A. simplex* (s.s.) located in the liver and mesentery, where the immune reactivity of the host seems to be stronger [60]. Conversely, a standard *GP* expression similar to the control was detected in larvae recovered in the host skeletal muscle, where the host’s immune reactivity seems to be weaker (Figure 2). Indeed, it is generally recognized that the parasite glycoproteins which occur abundantly on worms’ surface and in excretory/secretory (ES) products [61] are able to down-modulate the host immune response [61,62]. For instance, in the case of *Schistosoma mansoni* eggs, an omega-1 glycoprotein was found to promote Th-2 skewing of dendritic cells (DCs) and T cells during infection [63]. Previous studies have suggested that *Anisakis* spp. larvae secrete ES products, whose glycosylated components regulate the fundamental processes of antigen recognition, processing, and presentation [13]. The *Anisakis* glycoprotein would be able to interact with both innate and adaptive branches of the immune system in intermediate and accidental human hosts. Indeed, these molecules are involved in the regulation of the IgM response of the intermediate/paratenic fish host [64]. 

Lopienska-Biernat et al. [24] have recently described a pathway of genes coding proteins of trehalose and glycogen metabolism in *A. simplex* (s.l.). The trehalose has an important role in the energy storage, sugar circulation, glucose uptake in the absence of food, and protection during adverse environmental conditions [65,66]. Indeed, the trehalose is essential for survival of parasitic nematodes, e.g., *A. simplex* (s.s.) [67,68]. 

Third-stage larvae of *A. simplex* (s.l.) do not use external sources of glucose [68,69]. Indeed, even if the alimentary system is developed, there is no intake and assimilation of food, so the trehalose is necessary for the glucose storage and their survival without directly feeding [70,71,72]. In this study, no significantly high levels of *treh* gene in *A. simplex* (s.s.) larvae recovered from the liver and muscle, compared with control, were observed. However, significantly higher *treh* gene expression levels were detected in larvae recovered from the mesenteries (Figure 2). These results would reflect the necessity of the parasite L3 to transcribe the *treh* gene since trehalose appears to be indispensable to accumulate endogenous saccharide reserve compounds [72]. The *Anisakis* spp. larvae are typically surrounded by fibrous connective tissues generated by the fish host [3]; the capsule’s thickness formed around the larva seems to be related to the abundance of connective tissue in different infection sites in the host. Thus, it would occur that, where the connective tissue layers become more compact (i.e., mesenteries), the host’s immune system produces a thicker capsule around the larva, which remains “imprisoned” [73]. Capsules formation would lead to a major need of energy for the larva to contrast the host immune response, with a consequent increase of the *treh* gene transcripts. 

In parasitic nematodes, peptidase proteins represent a conspicuous percentage of expressed genes [74]. They are involved in host–parasite interactions [75] and, in many cases, constitute the main component of antigens, known for triggering immune responses of the host [76,77]. The metallopeptidases family is involved in the host tissue invasion by degrading the extracellular matrix. It has been also suggested that they conserve their proteolytic activity even after the action of the host antibodies [78,79]. Kim et al. [21] found that some metallopeptidases genes are differentially regulated in the *A. simplex* (s.l.) larvae transcriptome. In particular, the authors observed that the *nas 13* was up-regulated in L3 larvae in comparison with the L4 stage of the parasite species. Cavallero et al. [23] have found high transcripts of the metallopeptidases in the pharyngeal tissues of *Hysterothylacium aduncum* as well. These enzymes, in *Anisakis* spp. larvae, were considered to be involved in host tissue penetration [21]. In this study, a generalized up-regulation of *nas 13* was recorded in *A. simplex* (s.s.) larvae located in all the fish-host tissues, compared to the control (Figure 2). This finding highly supports a major role of the parasite’s peptidases in the fish host tissue invasion and migration. 

The role of ubiquitin-protein in *A. simplex* (s.l.) is not yet fully elucidated. It was hypothesized [80] that the ubiquitin–proteasome system could be involved in protein degradation and apoptosis in parasitic nematodes, as well as in the withstanding to the oxidative-stress. The ubiquitin pathway is also known to be involved in the survival of parasitic nematode *Trichinella spiralis* and in the development of the parasitic trematode *Schistosoma mansoni* [81]. Nematodes rely on proteomic plasticity to remodel themselves during periods of developmental change, to endure varying environmental conditions, and to respond to biotic and abiotic stressors. Considering *A. simplex* (s.s.) from the different tissues of *M. poutassou*, the gene *hyd* was apparently not differentially regulated compared to the control (Figure 2). This would indicate that the larvae lay in a stable developmental stage; i.e., they are not going to molt in the paratenic fish host. As a consequence, the parasite specimen at this stage does not need to regulate the internal state, usually mediated by the up-regulation of stress genes, such as the *hyd* [80].

Since a significantly higher *sfxn 2* expression in *A. simplex* (s.s.) L3 larvae compared to L4 was previously observed [21], the gene expression of the sideroflexin 2 in different sites of fish infection was here investigated. To date, it is known that the sideroflexins are mitochondrial multiple transmembrane proteins associated with the iron accumulation in the mitochondria [82]. In the present study, a down-regulation of *sfxn* 2 was recorded in *A. simplex* (s.s.) larvae removed from different fish tissues (Figure 2). However, because no information on the function of sideroflexin in parasitic nematodes is so far available, further studies are needed to elucidate the possible roles of the sideroflexin 2 in *A. simplex* (s.s.) larvae. 

## 5. Conclusions

*Anisakis* is an endoparasite exposed to changing life conditions, considering that its hosts vary between the poikilothermic crustaceans, heterothermic fish, and homeothermic marine mammals. However, the L4 stage and adults of *A. simplex* (s.l.) parasitize a more stable environment, such as the stomach of the cetacean definitive hosts, with consequent levels of transcripts more stable. Instead, the third-stage larvae differentially transcribe mRNAs, depending on different fish host tissue environments. Survival in an array of such different habitats must require costly molecular and biochemical adaptations. Natural selection should remove the behaviors that are not compensating benefits [84]. For example, during migration through the tissues, the larvae can become lost or killed by the fish host; migration seems to be not necessary for their development. 

Migration in different sites of infections of fish is probably an advantageous life-history strategy; however, the evolutionary factors responsible for the larval migration in the fish are poorly understood. 

The findings acquired during this study show that the relative gene expression levels of the seven target genes in the *A. simplex* (s.s.) larvae are linked to the infection site in the fish host, blue whiting. Indeed, different sites of infection influence the gene expression levels of *A. simplex* (s.s.) larvae. The genes encoding some of the present adaptive proteins seem to have protective properties for the parasite, facilitating host tissue migration (i.e., *nas 13*) and its survival (i.e., *TI, GL, hb*, *treh*) in the basically hostile target tissues of the fish host. Tissue migration is known from many different nematode orders and even other parasite phyla. Therefore, tissue migration has evolved many times and independently in the parasitic nematodes. 

An interesting question would be: why tissue migration has evolved at all? Why do larvae migrate over relatively long distances through a hostile environment instead of staying and developing in just one organ—the stomach or intestine, where the parasites first arrived? It could be assumed that tissue migration of *A. simplex* (s.s.) and *A. pegreffii* [8] is an adaptive and purpose-directed character, e.g., facilitating escape from host reactions (especially those in the flesh), and, similarly, that the host-induced encapsulation “protects”, in terms of isolating, the larvae, which remain in a “dormant” state until the larval transmission to the next host. 

## Figures and Tables

**Table 1:** Primers sets of target genes used in quantitative real-time PCR (qPCR) analysis.

Gene Locus	Sequence (5′ to 3′)	Ta/°C	Product Size (bp)	Reference
*TI*	CATGTGCCGATAAATGCGGG	57 °C	130	[25]
	CCCTGTGAGCATGCATCCTT			
*hb*	AAACATTCGACGCCTACACC	60 °C	108	[25]
	CATCGTGGTCTTCTCTGCGA			
*GP*	TATCGGAATGCGTGACTGCA	57 °C	130	[25]
	AGGCAGTTTCCATGGTGTATG			
*treh*	TCAGCAAGCATTTGAGTGAAGAGT	60 °C	117	[21]
	TAACATGATTGAAAACTTCGCAACA			
*nas 13*	AGCAATAGCAGCACGATGA	60 °C	131	[21]
	CTGCGGTAGCCAATGCTTTT			
*hyd*	CCATCCAGTGAAGAAGGATTCC	60 °C	116	[21]
	GAATAGAGCGGTAAGTAGAGCCTTGA			
*sfxn 2*	TTAGAATGGCGTTGAAGCAGTAGTAG	60 °C	130	[21]
	AGTATCGGTTCTGACCAGTTTTTTG			
*gpd*	CCCCTTCATCAACATCGACT	60 °C	152	[30]
	TCAGCTCCCCATTTGATTTC			

**Table 2:** Dunnett’s multiple comparison test for differences in gene expression level per target gene between larvae from different fish host tissues. Significance was fixed at *p* < 0.05 (* *p* < 0.05, ** *p* < 0.01, *** *p* < 0.001, **** *p* < 0.0001); *ns:* not significant.

Gene Locus	Liver vs. Mesentery	Liver vs. Muscle	Mesentery vs. Muscle
*TI*	****	****	*ns*
*hb*	*ns*	****	****
*GP*	*ns*	***	**
*treh*	*	*ns*	*
*nas 13*	*ns*	*	*ns*
*hyd*	*	*ns*	*ns*
*sfxn 2*	*	*ns*	****
